# Supplementary material for: An intranuclear bacterial parasite of deep-sea mussels expresses apoptosis inhibitors acquired from its host
Source: Nat Microbiol. 2024 Sep 6;9(11):2877–91. doi: 10.1038/s41564-024-01808-5 (PMC11521996; doi:10.1038/s41564-024-01808-5)
Supplement: Supplementary file 1 — Supplementary Figs. 1–3, Notes 1–6 and literature. [file 41564_2024_1808_MOESM1_ESM.pdf]

nature microbiology

Supplementary information https://doi.org/10.1038/s41564-024-01808-5

An intranuclear bacterial parasite of deep-sea mussels expresses apoptosis inhibitors acquired from its host

In the format provided by the authors and unedited

Supplementary notes

Supplementary Note 1 - Description of two "*Ca.* Endonucleobacter" species

[1] referred to the "*Ca*. Endonucleobacter" from nine bathymodoline species collectively as "*Ca*. Endonucleobacter bathymodioli" based on high similarities between their 16S rRNA genes (>98%). In our study using whole genome sequencing, average nucleotide identity (ANI) between the "*Ca*. Endonucleobacter" from *Gigantidas childressi* and *Bathymodiolus puteoserpentis* was 84.3%. In accordance with recent studies on ANI values of >95% delineating bacterial species [2,3], we named the intranuclear parasite from *G. childressi* "*Ca.* Endonucleobacter childressi" and from *B. puteoserpentis* "*Ca.* Endonucleobacter puteoserpentis" (for genome characteristics see **Supplementary Information Table 2)**. Our phylogenomic analysis and the AAI analysis placed them as a separate genus within the family *Endozoicomonaceae* (**Figure 1, Supplementary Figure 2**).

Supplementary Note 2 - Identification of inhibitors of apoptosis

A preliminary comparison of "*Ca.* Endonucleobacter" genomes with the JGI pipeline [4] and the RAST pipeline [5] suggested the presence of IAPs in their genomes. To substantiate these findings, we generated HMM profiles of 48 publicly available metazoan and viral BIR-containing proteins (BIRPs). A hmmsearch (http://hmmer.org/) was performed against the profiles using default thresholds (E-value 1x10E^{-3}) to identify and annotate the IAPs in "*Ca.* Endonucleobacter". To differentiate between BIRPs and bona fide IAPs in the bacterial genomes, we identified the protein domains of hmmsearch hits with the NCBI protein domain search platform (https://www.ncbi.nlm.nih.gov/Structure/cdd/wrpsb.cgi). Only sequences with a RING domain and at least one BIR-repeat domain were considered to be bona fide IAPs (**Extended Data Fig. 5**), while ORFs with at least one BIR-repeat but without a RING

domain were classified as BIRPs. To verify that IAPs were encoded in the "*Ca.* Endonucleobacter" chromosome and not the result of contamination with mussel sequences, we checked the location of "*Ca.* Endonucleobacter" IAPs using the graphical representation of its genome in Bandage to ensure that the IAPs were fully integrated in the bacterial chromosome [6] (**Supplementary Fig. 1**).

Supplementary Note 3 – "*Ca.* Endonucleobacter" may manipulate its host cell cytoskeleton

To understand how "*Ca.* Endonucleobacter" colonizes and manipulates its host beyond IAPs, we analyzed its virulence arsenal. Despite its reduced genome compared to closes relatives, "*Ca*. E. childressi" encoded and expressed a broad range of secretion systems. It encoded a T1SS and a T1SS-dependent RTX adhesin as well as the general secretory (Sec) pathway and the twin-arginine translocation (Tat) pathway, with most genes expressed and the T1SS-dependent RTX adhesin also present in the proteome (**Supplementary Information Table 9, 10**). "*Ca*. E. childressi" also encoded a T3SS, and upregulated most T3SS factors towards the end of the infectious cycle (**Supplementary Information Table 17**).

"Ca. E. childressi" encoded and expressed virulence factors with a T3SS-specific N-terminal signal peptide, indicating their secretion. Two of these effectors were annotated as the *Shigella*-like factor IpgD (inositol phosphate phosphatase), and both were present in the bulk transcriptome (**Fig. 2; Supplementary Information Table 3, 4**). In the enteropathogen *Shigella flexneri*, IpgD is injected via the T3SS into host cells, where it interferes with the actin network of host cells [7]. If IpgD functions similarly in "*Ca.* E. childressi", it could reduce the cytoskeletal stress caused by the massive volume increase of the nucleus, thereby maintaining the functioning of host cells and delaying apoptosis. This hypothesis is supported by the fact that *IpgD* expression increased in the mid and late infection stages (**Fig. 3; Supplementary Information Table 7**), when the volume of the infected nuclei increased the most. Overall, our

analyses show that “*Ca.* E. childressi” uses a broad range of secretion systems and effectors to interfere with host cytoskeletal processes. Together with apoptosis inhibition via IAPs, these strategies likely work synergistically to ensure that “*Ca.* E. childressi” can replicate to such high numbers inside the nucleus, the essential 'command center' for host cell viability.

Supplementary Note 4 - Nesprin-1 as reporter of nuclear deformation

To study the host processes that might activate apoptosis, we investigated host factors that were upregulated during “*Ca.* Endonucleobacter” infection. The host cell consistently upregulated nesprin-1 in all three infection stages (**Fig. 3; Supplementary Information Table 8**). Nesprins are transmembrane proteins in the nuclear envelope that are part of the nucleoskeleton component of the LINC (linker of nucleoskeleton and cytoskeleton) complex that connects the nucleus with the cytoplasm [8]. Nesprin-1 plays a key role as a biomechanical reporter for nuclear position and morphology [9], as well as in the DNA damage response pathway [10]. Furthermore, recent studies have shown that Nesprin-2 have pro-apoptotic activity via the LINC complex [11]. We interpret the upregulation of nesprin-1 in infected *G. childressi* cells as an apoptotic response to the nuclear expansion induced by “*Ca.* E. childressi”.

Supplementary Note 5 - Repeated host-to-symbiont HGT in *Endozoicomonadaceae*

“Ca. Endonucleobacter” and *Endozoicomonas ascidiicola* IAPs are more closely related to IAPs from their animal hosts than to each other, and fall into clades separated by IAPs from other animals, indicating that eukaryote-to-bacteria HGT of IAPs occurred more than once in the *Endozoicomonadaceae* (**Fig. 4**). IAPs have ankyrin repeats (ANKs) in their RING domain, a protein motif that is widespread and common in eukaryotes. Like “*Ca.* Endonucleobacter”, *Endozoicomonas* genomes are enriched in ANKs and mobile genetic elements (insertion sequences), suggesting that these genomic features are a common trait of the family *Endozoicomonadaceae*

(**Supplementary Information Table 2)** [12–15]. Mobile elements are known to promote HGT in microorganisms [16], and might have facilitated repeated acquisition of IAPs and other animal host genes, enriching *Endozoicomonadaceae* genomes with these eukaryotic-like domains. Given that eukaryotic-like domains are enriched in bacteria associated with eukaryotes, together with studies showing that these proteins modulate interactions between bacteria and their eukaryotic hosts, it is possible that genomic enrichment in ANKs and other eukaryotic-like proteins enabled the enormous versatility of *Endozoicomonadaceae* in associating with a wide range of animals from marine environments around the world [17–21].

Supplementary figures

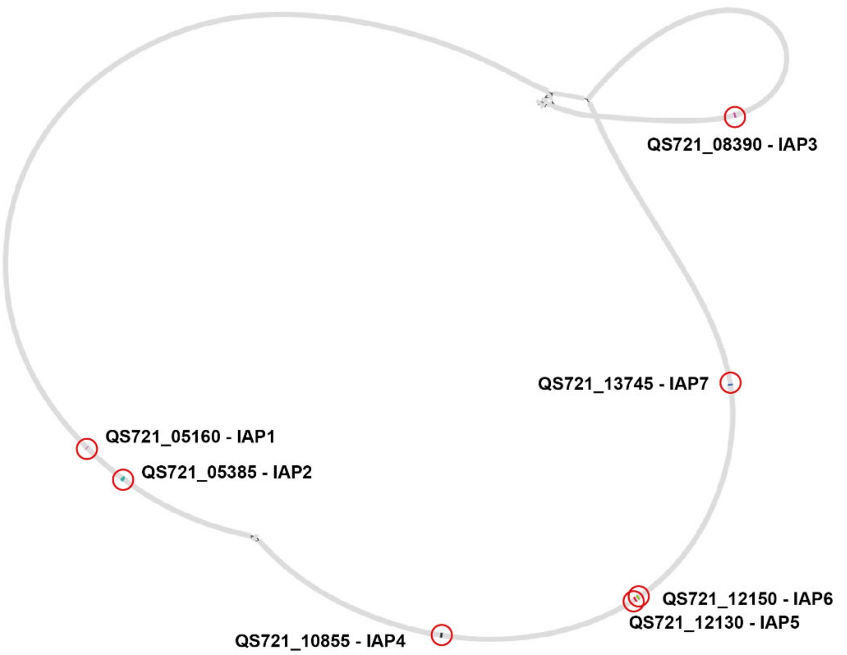

Supplementary Figure 1 | All seven IAPs encoded by "*Ca.* E. childressi" were located on its chromosomal contigs. Graphic representation of "*Ca.* E. childressi" genome assembly (Bandage). Red circles show the location of IAPs within their respective contigs.

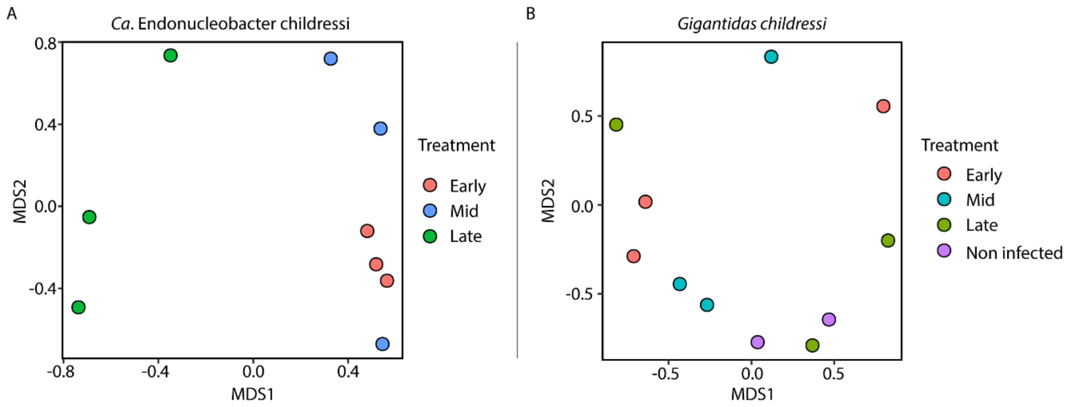

Supplementary Figure 2 | Sample variation of the LMD-transcriptomic pipeline. NDMS plot showing the variation within the LMD dataset for (A) "*Ca.* Endonucleobacter childressi" on the left and (B) the *G. chilressi* host cell on the right.

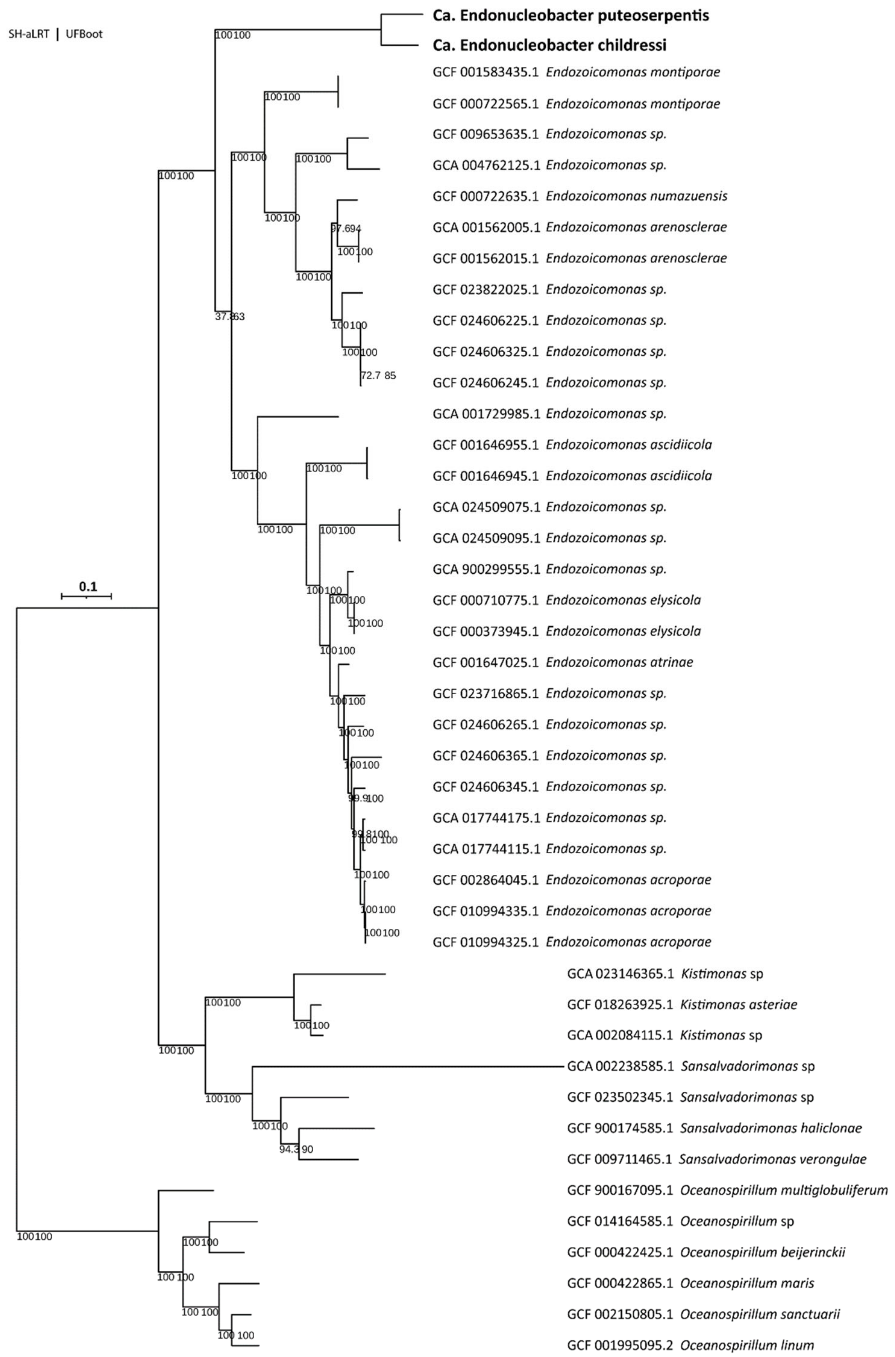

Supplementary Figure 3 | Phylogenomic analysis using 172 conserved marker genes shared between shared between the two "*Ca.* Endonucleobacter" genomes and those of 42 closely related *Endozoicomonaceae*. Genes were identified and aligned with the GToTree pipeline, the tree calculated with IQTREE, and branch support (1000 replicates) was calculated with both SH-aLRT and UFBoot. Six *Oceanospirillum* genomes were used to root the tree. Scale bars indicate substitutions per site.

Supplementary literature

1. Zielinski, F. U. *et al.* Widespread occurrence of an intranuclear bacterial parasite in vent and seep bathymodiolin mussels. *Environ. Microbiol.* **11**, 1150–1167 (2009).
2. Barco, R. A. *et al.* A genus definition for Bacteria and Archaea based on a standard genome relatedness index. *MBio* **11**, (2020).
3. Olm, M. R. *et al.* Consistent metagenome-derived metrics verify and delineate bacterial species boundaries. *mSystems* **5**, 10.1128/msystems.00731-19 (2020).
4. Nordberg, H. *et al.* The genome portal of the Department of Energy Joint Genome Institute: 2014 updates. *Nucleic Acids Res.* **42**, D26–D31 (2014).
5. Aziz, R. K. *et al.* The RAST server: Rapid annotations using subsystems technology. *BMC Genomics* **9**, 75 (2008).
6. Wick, R. R., Schultz, M. B., Zobel, J. & Holt, K. E. Bandage: Interactive visualization of *de novo* genome assemblies. *Bioinformatics* **31**, 3350–3352 (2015).
7. Köseoğlu, V. K., Jones, M. K. & Agaisse, H. The type 3 secretion effector IpgD promotes *S. flexneri* dissemination. *PLOS Pathog.* **18**, e1010324 (2022).
8. Bouzid, T. *et al.* The LINC complex, mechanotransduction, and mesenchymal stem cell function and fate. *J. Biol. Eng.* **13**, 68 (2019).
9. Zhang, J. *et al.* Nesprin 1 is critical for nuclear positioning and anchorage. *Hum. Mol. Genet.* **19**, 329–341 (2010).
10. Sur, I., Neumann, S. & Noegel, A. A. Nesprin-1 role in DNA damage response. *Nucleus* **5**, 173–191 (2014).
11. Lindenboim, L., Zohar, H., Gundersen, G. G., Worman, H. J. & Stein, R. LINC complex protein nesprin-2 has pro-apoptotic activity via Bcl-2 family proteins. *Cell Death Discov.* **10**, 29 (2024).
12. Neave, M. J. *et al.* Differential specificity between closely related corals and abundant *Endozoicomonas* endosymbionts across global scales. *ISME J.* **11**, 186–200 (2017).
13. Alex, A. & Antunes, A. Comparative genomics reveals metabolic specificity of *Endozoicomonas* isolated from a marine sponge and the genomic repertoire for host-bacteria symbioses. *Microorganisms* **7**, 635 (2019).
14. Pogoreutz, C. *et al.* Coral holobiont cues prime *Endozoicomonas* for a symbiotic lifestyle. *ISME J.* **16**, 1883–1895 (2022).
15. Ide, K. *et al.* Targeted single-cell genomics reveals novel host adaptation strategies of the symbiotic bacteria *Endozoicomonas* in *Acropora tenuis* coral. *Microbiome* **10**, 220 (2022).
16. Haudiquet, M., de Sousa, J. M., Touchon, M. & Rocha, E. P. C. Selfish, promiscuous and sometimes useful: How mobile genetic elements drive horizontal gene transfer in microbial

populations. *Philos. Trans. R. Soc. B Biol. Sci.* **377**, (2022).

17. Ding, J.-Y., Shiu, J.-H., Chen, W.-M., Chiang, Y.-R. & Tang, S.-L. Genomic insight into the host–endosymbiont relationship of *Endozoicomonas montiporae* CL-33T with its coral host. *Front. Microbiol.* **7**, (2016).
18. Gomez-Valero, L., Rusniok, C., Cazalet, C. & Buchrieser, C. Comparative and functional genomics of *Legionella* identified eukaryotic like proteins as key players in host-pathogen Interactions. *Front. Microbiol.* **2**, 1–20 (2011).
19. Nguyen, M. T. H. D., Liu, M. & Thomas, T. Ankyrin-repeat proteins from sponge symbionts modulate amoebal phagocytosis. *Mol. Ecol.* **23**, 1635–1645 (2014).
20. Maire, J. *et al.* Colocalization and potential interactions of *Endozoicomonas* and chlamydiae in microbial aggregates of the coral *Pocillopora acuta*. *Sci. Adv.* **9**, eadg0773 (2023).
21. Neave, M. J., Apprill, A., Ferrier-Pagès, C. & Voolstra, C. R. Diversity and function of prevalent symbiotic marine bacteria in the genus *Endozoicomonas*. *Appl. Microbiol. Biotechnol.* **100**, 8315–8324 (2016).
