# Supplementary material for: An intranuclear bacterial parasite of deep-sea mussels expresses apoptosis inhibitors acquired from its host
Source: Nat Microbiol. 2024 Sep 6;9(11):2877–91. doi: 10.1038/s41564-024-01808-5 (PMC11521996; doi:10.1038/s41564-024-01808-5)
Supplement: Supplementary file 2 — Reporting Summary [file 41564_2024_1808_MOESM2_ESM.pdf]

nature portfolio

Corresponding author(s): Nicole Dubilier, Nikolaus Leisch

Last updated by author(s): Jul 30, 2024

Reporting Summary

Nature Portfolio wishes to improve the reproducibility of the work that we publish. This form provides structure for consistency and transparency in reporting. For further information on Nature Portfolio policies, see our Editorial Policies and the Editorial Policy Checklist.

Statistics

For all statistical analyses, confirm that the following items are present in the figure legend, table legend, main text, or Methods section.

n/a	Confirmed	
☐	☒	The exact sample size (*n*) for each experimental group/condition, given as a discrete number and unit of measurement
☐	☒	A statement on whether measurements were taken from distinct samples or whether the same sample was measured repeatedly
☒	☐	The statistical test(s) used AND whether they are one- or two-sided<br>*Only common tests should be described solely by name; describe more complex techniques in the Methods section.*
☒	☐	A description of all covariates tested
☒	☐	A description of any assumptions or corrections, such as tests of normality and adjustment for multiple comparisons
☐	☒	A full description of the statistical parameters including central tendency (e.g. means) or other basic estimates (e.g. regression coefficient) AND variation (e.g. standard deviation) or associated estimates of uncertainty (e.g. confidence intervals)
☒	☐	For null hypothesis testing, the test statistic (e.g. *F*, *t*, *r*) with confidence intervals, effect sizes, degrees of freedom and *P* value noted<br>*Give P values as exact values whenever suitable.*
☒	☐	For Bayesian analysis, information on the choice of priors and Markov chain Monte Carlo settings
☒	☐	For hierarchical and complex designs, identification of the appropriate level for tests and full reporting of outcomes
☒	☐	Estimates of effect sizes (e.g. Cohen's *d*, Pearson's *r*), indicating how they were calculated

Our web collection on statistics for biologists contains articles on many of the points above.

Software and code

Policy information about availability of computer code

Data collection	No specialized software was used for data collection.
Data analysis	RAST v2.0: https://rast.nmpdr.org/<br>JGI annotation server: https://genome.jgi.doe.gov/portal/<br>NCBI BLAST v2.10.1<br>Pathway Tools v13.0<br>SignalP v6.0<br>MAFFT v7.407<br>MAFFT v7.471<br>IQTREE v1.6.12<br>xT microscope control software v6.2.6.3123<br>cRAP protein sequence database v2012.01.01: http://www.thegpm.org/crap/<br>Proteome Discoverer v2.2.0.388<br>BBMap v.38.90, including BBDuk, BBNorm https://sourceforge.net/projects/bbmap/<br>FeatureCounts v1.6.3<br>Aldex2 v3.11<br>PhyloFlash v3.3<br>Spades v3.7<br>Gbtools v2.6.0<br>Bandage v0.8.1<br>CheckM v1.0.18

ngmlr v0.2.7
CANU v2.0
Unicycler v0.4.8
SILVA database v123: https://www.arb-silva.de/
Kallisto v.0.44.0
Trinity v.2.10.0
BUSCO v.4.1.2
MEGAN v.6.16.4
NCBI protein domain analysis, CDD v.3.21-62456 PSSMs: https://www.ncbi.nlm.nih.gov/Structure/cdd/wrpsb.cgi
Fiji 1.52v
Clustal Omega v1.2.2
ISEScan v1.7.2.3
Olympus cellSens Dimension software v1.18
ZEN software v14.0.1.201
Adobe Photoshop / Adobe Illustrator v12
GToTree v1.8.4
HHMMER3 v3.1b2
HHMMER3 v3.4
Muscle 5.1.linux64
TrimAl v1.4.rev15
Prodigal v2.6.3
Genome Taxonomy Database (GTDB), Release 09-RS220
FastTree 2 v2.1.11
GNU Parallel v20240122
IQTREE 2.3.0
RStudio v1.3.1093
vegan v2.6-4

For manuscripts utilizing custom algorithms or software that are central to the research but not yet described in published literature, software must be made available to editors and reviewers. We strongly encourage code deposition in a community repository (e.g. GitHub). See the Nature Portfolio guidelines for submitting code & software for further information.

Data

Policy information about availability of data

All manuscripts must include a data availability statement. This statement should provide the following information, where applicable:

- Accession codes, unique identifiers, or web links for publicly available datasets
- A description of any restrictions on data availability
- For clinical datasets or third party data, please ensure that the statement adheres to our policy

The metagenomic and metatranscriptomic raw reads and assembled symbiont genomes are available in The National Center for Biotechnology Information (NCBI) under BioProject Accession Number PRJNA979916. The annotated genomes of both “Ca. Endonucleobacter” species, as used in this study, the host transcriptomes and their annotations, and the HMM profiles used to identify IAPs and the microscope data used to generate the figures are available in the ZENODO repository under DOI: 10.5281/zenodo.11086255.
The mass spectrometry metaproteomics data and protein sequence database were deposited in the ProteomeXchange Consortium via the PRIDE partner repository with the dataset identifier PXD020317. The genomes of "Ca. Endonucleobacter childressi" and "Ca. Endonucleobacter puteoserpentis" generated in this study were submitted to NCBI under the accession numbers GCA030674875.1 and GCA030674915.1, respectively.
For the construction of the phylogenetic tree in Figure 1, genomes were downloaded from NCBI ftp://ftp.ncbi.nlm.nih.gov/genomes/all/ with accession numbers listed in Figure 1. For the construction of the phylogenetic tree in Figure 4, protein sequences were downloaded from NCBI https://ncbi.nlm.nih.gov/ with accession numbers listed in Figure 4.

Research involving human participants, their data, or biological material

Policy information about studies with human participants or human data. See also policy information about sex, gender (identity/presentation), and sexual orientation and race, ethnicity and racism.

Reporting on sex and gender	N/A
Reporting on race, ethnicity, or other socially relevant groupings	N/A
Population characteristics	N/A
Recruitment	N/A
Ethics oversight	N/A

Note that full information on the approval of the study protocol must also be provided in the manuscript.

Field-specific reporting

Please select the one below that is the best fit for your research. If you are not sure, read the appropriate sections before making your selection.

☐ Life sciences ☐ Behavioural & social sciences ☒ Ecological, evolutionary & environmental sciences

For a reference copy of the document with all sections, see nature.com/documents/nr-reporting-summary-flat.pdf

Ecological, evolutionary & environmental sciences study design

All studies must disclose on these points even when the disclosure is negative.

Study description	Symbiotic deep-sea mussles of the genus Bathymodiolus and Gigantidas were analyzed to study the association with intra-nuclear bacteria
Research sample	Mussels were collected with remotely operated vehicles during two research cruises. Onboard, the mussels' gills were dissected, preserved, and stored for further processing. The symbiont housing organ (gill) was subjected to metagenome and metatrancriptome sequencing, metaproteomics and FISH analyses.
Sampling strategy	Mussels were sampled with a net from their natural habitat. Sampling of individuals depends on fieldwork conditions. Sampling sizes are sufficient for analyses performed in study
Data collection	none
Timing and spatial scale	Gigantidas childressi mussels were collected with the ROV Hercules during the RV Meteor Nautilus NA-58 cruise to the Gulf of Mexico in May 2015 at the Mississippi Canyon site (MC853, 28º07′ N; -089º08′ W) and the Green Canyon site (GC234, 27º45′ N; -091º13′ W) at water depths of 1,070 and 540 m, respectively. B. puteoserpentis mussels were collected with the ROV MARUM-QUEST during the Meteor M126 cruise to the Mid-Atlantic Ridge in April 2016 from the Logatchev vent field (Irina-II smoker, 14º45′ N; -044º59′ W) at a water depth of 3,036 m.
Data exclusions	no data were excluded
Reproducibility	Using the deposited raw sequencing, proteomic and imaging data, the data analyses that were performed in this study can be easily and repeatedly reproduced.
Randomization	Not relevant. To study the intra-nuclear association, samples were screened for the presence of the intra-nuclear parasite.
Blinding	Blinding was not performed because it was not relevant to this study. This study was an exploratory survey without a priori expectations that would influence the analyses.

Did the study involve field work? ☒ Yes ☐ No

Field work, collection and transport

Field conditions	Deep-sea sampling at hydrothermal vents. Temperatures of mussel occurences were usually between 4 and 10 °C (for those where measurement was available).
Location	Gigantidas childressi mussels were collected with the remotely operated vehicle (ROV) Hercules during the RV Meteor Nautilus NA-58 cruise to the Gulf of Mexico in May 2015 at the Mississippi Canyon site (MC853, 28º07′ N; -089º08′ W) and the Green Canyon site (GC234, 27º45′ N; -091º13′ W) at water depths of 1,070 and 540 m, respectively. B. puteoserpentis mussels were collected with the ROV MARUM-QUEST during the Meteor M126 cruise to the Mid-Atlantic Ridge in April 2016 from the Logatchev vent field (Irina-II smoker, 14º45′ N; -044º59′ W) at a water depth of 3,036 m.
Access & import/export	Material used in this study were collected during German and US research cruises. Animals were collected from the deep/sea floor using remote operated vehicles. Bathymodiolus mussels are non-commercial and are not subjected to CITES or any other international regulations. Import permissions into Germany were granted by German authorities, where necessary.
Disturbance	All sampling adhered to the InterRigdeCode of conduct of work at hydrothermal vents (https://www.interridge.org/irstatement)

Reporting for specific materials, systems and methods

We require information from authors about some types of materials, experimental systems and methods used in many studies. Here, indicate whether each material, system or method listed is relevant to your study. If you are not sure if a list item applies to your research, read the appropriate section before selecting a response.

Materials & experimental systems

n/a	Involved in the study	
☒	☐	Antibodies
☒	☐	Eukaryotic cell lines
☒	☐	Palaeontology and archaeology
☐	☒	Animals and other organisms
☒	☐	Clinical data
☒	☐	Dual use research of concern
☒	☐	Plants

Methods

n/a	Involved in the study	
☒	☐	ChIP-seq
☒	☐	Flow cytometry
☒	☐	MRI-based neuroimaging

Animals and other research organisms

Policy information about studies involving animals; ARRIVE guidelines recommended for reporting animal research, and Sex and Gender in Research

Laboratory animals	No laboratory animals were used in this study.
Wild animals	Mussels of the species Gigantidas childressi and Bathymodiolus puteoserpentis were collected from hydrothermal vent and cold seep mussel fields with remotely operated vehicles operated from board of research vessels using nets. Mussels were transported in ambient water (4°C - 8°C) in a temperature isolated container to the surface where they were dissected and the tissue preserved for different experiments.
Reporting on sex	Sex was not considered for this study.
Field-collected samples	Adult mussels of the species Gigantidas childressi and Bathymodiolus puteoserpentis, of unknown age, with a shell length of 8-10cm were collected from hydrothermal vent and cold seep mussel fields with remotely operated vehicles operated from board of research vessels, using nets. Mussels were transferred into a temperature-isolated box full of ambient seawater, that kept them at ambient temperature and in the dark. They were transported to the surface where they were immediately dissected and the tissue preserved for different experiments.
Ethics oversight	Work on these mussels is not subjected to a approval by an ethics committee

Note that full information on the approval of the study protocol must also be provided in the manuscript.

Plants

Seed stocks	No plants were used in this study
Novel plant genotypes	No plants were used in this study
Authentication	No plants were used in this study
